# Grafting of Maleic Anhydride onto Poly(vinylidene fluoride) Using Reactive Extrusion

**DOI:** 10.3390/molecules28052246

**Published:** 2023-02-28

**Authors:** Cuichuan Zhang, Zhiqiang Lu, Bin Wu, Shu-Dong Jiang, Jiasheng Qian

**Affiliations:** Key Laboratory of Environment-Friendly Polymeric Materials of Anhui Province, School of Chemistry and Chemical Engineering, Anhui University, Hefei 230601, China

**Keywords:** PVDF, MAH, stem grafting, melt extrusion

## Abstract

Poly(vinylidene fluoride) was grafted with maleic anhydride through reactive extrusion by using diisopropyl benzene peroxide as an initiator and 9-vinyl anthracene as a stabilizer. Effects of various parameters on grafting degree were investigated including the amounts of monomer, initiator and stabilizer. The maximum extent of grafting achieved was 0.74%. The graft polymers were characterized using FTIR, water contact angle, thermal, mechanical and XRD studies. Improved hydrophilic and mechanical properties were observed for graft polymers.

## 1. Introduction

Fluorinated polymers are niche macromolecules that exhibit unique and remarkable attributes, largely due to the properties of fluorine (including large electronegativity, low polarizability and small van-der-Waals radius (1.32 Å)) and the strong C–F bonds (485 kJ·mol^−1^) [1,2]. As a kind of semi-crystalline fluoropolymer, polyvinylidene fluoride (PVDF) has received wide attention in both academia and industry, as it shows outstandingly high chemical stability to solvents, acids and bases, weather resistance to oxidative and ultraviolet decomposition, as well as excellent ferroelectric, piezoelectric and other unique electrical properties [3,4,5,6,7]. However, PVDF is an inert polymer that lacks the presence of reactive functionality, and therefore shows poor adhesive properties with substrates and inferior compatibility with other polymers; this limits the application of PVDF in technological fields.

Fluorinated polymers are niche macromolecules that exhibit unique and remarkable attributes, largely due to the properties of fluorine (including large electronegativity, low polarizability and small van-der-Waals radius (1.32 Å)) and the strong C–F bonds (485 kJ·mol^−1^) [1,2]. As a kind of semi-crystalline fluoropolymer, polyvinylidene fluoride (PVDF) has received wide attention in both academia and industry, as it shows outstandingly high chemical stability to solvents, acids and bases, weather resistance to oxidative and ultraviolet decomposition, as well as excellent ferroelectric, piezoelectric and other unique electrical properties [3,4,5,6,7]. However, PVDF is an inert polymer that lacks the presence of reactive functionality, and therefore shows poor adhesive properties with substrates and inferior compatibility with other polymers; this limits the application of PVDF in technological fields.

Recently, efforts have been made to improve the performance of PVDF by blending PVDF with other polymers/nanomaterials, chemically grafting functional molecules onto PVDF or copolymerizing it with vinylidene fluoride (VDF) and other monomers [8,9,10,11,12,13,14,15,16,17,18,19,20]. Chen et al. improved the interface compatibility between polypropylene (PP) and PVDF by using a styrene-maleic-anhydride copolymer as a compatibilizer [8]. By intermixing PVDF with maleic anhydride (MAH), Ye et al. found that MAH acts as a physical crosslinking point for PVDF macromolecules [9]. Naruke et al. found that the mixing ratio-dependent melting point depression exhibited by polymethyl methacrylate (PMMA)/PVDF blends is due to the size of their interface region, and that the complex thermophysical behavior of the solution-casting blends is caused by the presence of ultrafine PVDF crystals [10]. Tang et al. fabricated antifouling PVDF hollow fiber membranes through a zwitterionic graft copolymerization method [11]. Ameduri et al. summarized 2-trifluoromethacrylic acid, ω-trifluorovinyl and other macromonomers and their potential to react with VDF (including copolymerization kinetics), the properties of the resulting copolymers and their applications in daily life. At the same time, the effect of the crosslinking of this VDF copolymerization with special functions on this kind of fluorinated material is described [12]. Furthermore, the preparation and use of VDF copolymers are summarized [18]. Qiu et al. report the surface modification of PVDF microporous membranes by heat-induced grafting copolymerization with MAH/styrene (St) with a supercritical carbon dioxide (SCCO_2_) solvent and a carrier agent. SCCO_2_ can accelerate the mass transfer of the monomer in the polymer group, and then promote the grafting copolymerization at the membrane surface and in membrane pores. It was shown that significant and permanent hydrophilicity was obtained after grafting SMA and that the SMA-based PVDF membrane surface had excellent biocompatibility [19]. Although the studies of PVDF have gradually deepened, there are still few reports on a simple, fast and scalable approach to graft modified PVDF.

The reactive extrusion process is favorable due to its advantages such as fast reaction, continuous process, low cost and accessibility to industrialization, and has gained considerable interest compared to other grafting methods [21,22,23,24,25]. Iqbal et al. [21] modified low-density polyethylene with MAH by using benzoyl peroxide (BPO) as an initiator in a co-rotating twin-screw extruder. With the increase in BPO and MAH content, the MAH-grafting degree increased first and then decreased when reaching a certain value. Ismail prepared an ethylene-propylene-diene monomer (EPDM)-g-MAH by melting and mixing. The presence of EPDM-g-MAH can enhance interfacial interaction between EPDM matrix and bentonite [22]. Choi et al., fabricated MAH-EPDM grafted MWCNTs by reactive extrusion to enhance interfacial adhesion and mechanical properties of PP/MAH-EPDM composite [23]. Simonetti reported that the functional modified polyamide 6 (PA6) was performed through Michael addition between divinyl phenyl phosphine oxide and piperazine during the melt extrusion process of PA6 [24]. Through introducing silane-grafted lignin into polybutylene adipate-co-terephthalate (PBAT), a network structure between PBAT and modified lignin was formed by the reactive extrusion, which can enhance mechanical and biodegradable properties [25]. Arkema has already marketed PVDF-g-MAH, and patents have been reported for the fluoropolymer grafting method [26].

**Scheme 1:**
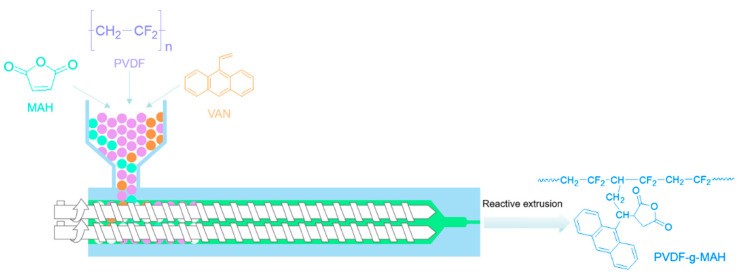
The preparation of PVDF-g-MAH through reactive extrusion.

Herein, PVDF was grafted with MAH through reactive extrusion by using diisopropyl benzene peroxide (DCP) as an initiator and 9-vinyl anthracene (VAN) as a free-radical stabilizer, as shown in Scheme 1. Due to the introduction of an anhydride group of MAH onto the PVDF side chain, PVDF is normalized to improve the hydrophilicity of PVDF and its compatibility with other substances.

## 2. Results

### 2.1. FTIR Analysis

**Figure 1:**
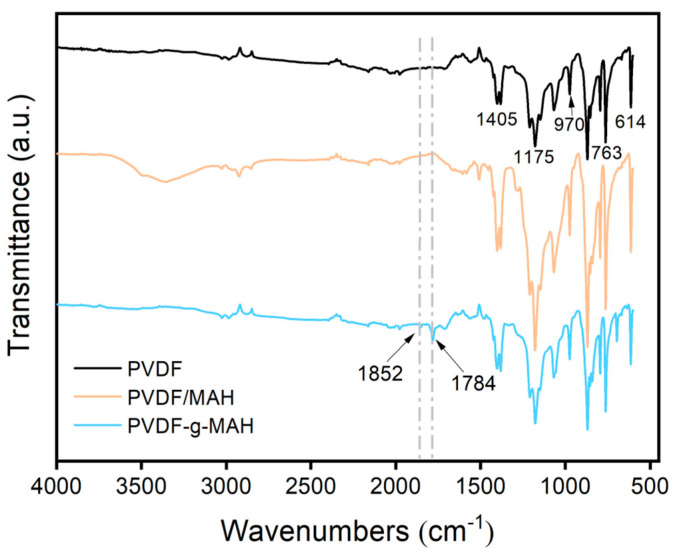
FTIR spectra of pure PVDF, PVDF/MAH and PVDF-g-MAH (G = 0.63%).

The FTIR results of pure PVDF, PVDF/MAH and PVDF-g-MAH are shown in Figure 1. For the pure PVDF, the strong absorption bands at 1405 cm^−1^ and 1175 cm^−1^ are attributed to the -CH_2_ deformation vibration and the -CF_2_ stretching vibration The visible absorption bands at 970 cm^−1^, 763 cm^−1^ and 614 cm^−1^ are typical characteristics of the PVDF nonpolar α phase [27]. Additionally, the position of PVDF absorption peaks in the PVDF/MAH and PVDF-g-MAH is similar to that of pure PVDF. In contrast to the pure PVDF and PVDF/MAH, two new absorption peaks can be observed at 1852 cm^−1^ and 1784 cm^−1^, corresponding to the symmetric and asymmetric stretching of the two C=O groups in the resulting succinic anhydride group [27]. It therefore indicates that MAH was successfully grafted onto the PVDF molecule chains.

### 2.2. Wetting Properties

**Figure 2:**
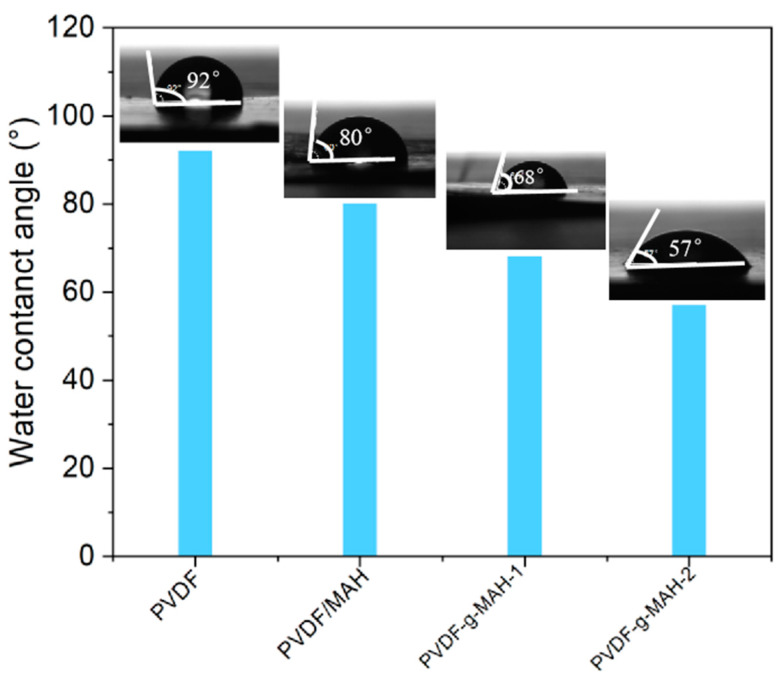
Water contact angles of pure PVDF, PVDF/MAH, PVDF-g-MAH-1 and PVDF-g-MAH-2.

Figure 2 shows the water contact angles of PVDF, PVDF/MAH and PVDF-g-MAH. Pure PVDF demonstrates hydrophobic properties due to its low surface energy. After blending with MAH containing a hydrophilic group, the hydrophilicity of PVDF exhibits a certain improvement. However, this improvement is limited, which is due to MAH being mostly dissolved during purification. Compared to PVDF/MAH, the hydrophilicity of PVDF-g-MAH-1 has an obvious increase. Moreover, the increase in the VAN content can better promote the grafting MAH onto PVDF, thus improving the hydrophilicity of PVDF more significantly, with the water contact angle dropping down to 57° (PVDF-g-MAH-2). The surface contact angle results further suggest that the preparation of PVDF-g-MAH was successful.

### 2.3. Grafting Degree

**Figure 3:**
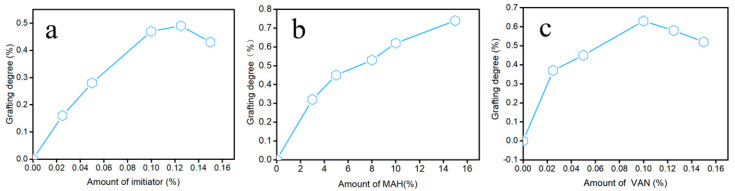
Effect of initiator concentration (**a**), MAH concentration (**b**) and VAN concentration (**c**) on grafting degree.

**Scheme 2:**
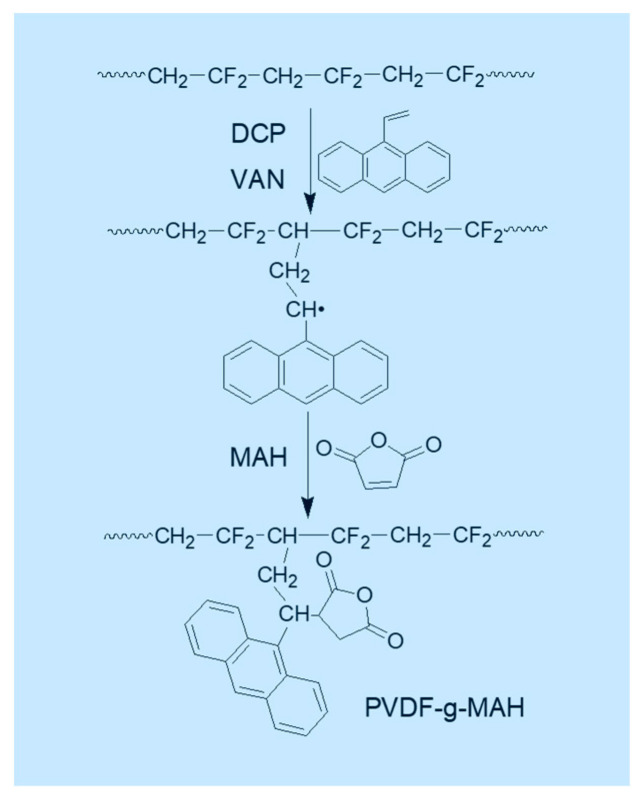
Reaction mechanism of free-radical-initiated grafting of MAH onto PVDF in the presence of VAN.

In order to optimize the conditions for grafting of MAH onto PVDF, a series of contrast experiments were carried out by adjusting the amount of DCP, MAH and VAN. Figure 3 shows the changing trend of MAH-grafting degree under different variables. The effect of initiator concentration on grafting degree is illustrated in Figure 3a. The observed trend is typical for the graft polymerization reaction occurring via chain transfer. The initial increase in the percent grafting is caused by an increase in the concentration of radicals formed through the decomposition of the initiator. Thus, the higher the concentration of radicals, the higher the chain transfer to the polymer backbone and the higher the percent grafting. Furthermore, an increase in initiator concentration decreases the average molecular weight of the side chains because of mutual termination reactions. As shown in Figure 3b, increasing the amount of MAH gives rise to an increase in the level of g-MAH, indicating that the content of MAH has a direct connection to the grafting degree. The role of VAN on the grafting degree of MAH onto PVDF is examined. Figure 3c shows that a higher concentration of VAN in the grafting system resulted in an increase in the grafting level. VAN is more resonance stabilized with the aromatic ring, leading to the observed continuous rate of the graft-polymerization reaction that results in an ultimately higher level of grafting. Thus, VAN acts as a promoter for the grafting reaction of MAH via an in situ co-grafting process resulting in a polymer containing highly grafted MAH. The possible grafting route is shown in Scheme 2.

### 2.4. Rheological Properties of PVDF, PVDF/MAH and PVDF-g-MAH

**Figure 4:**
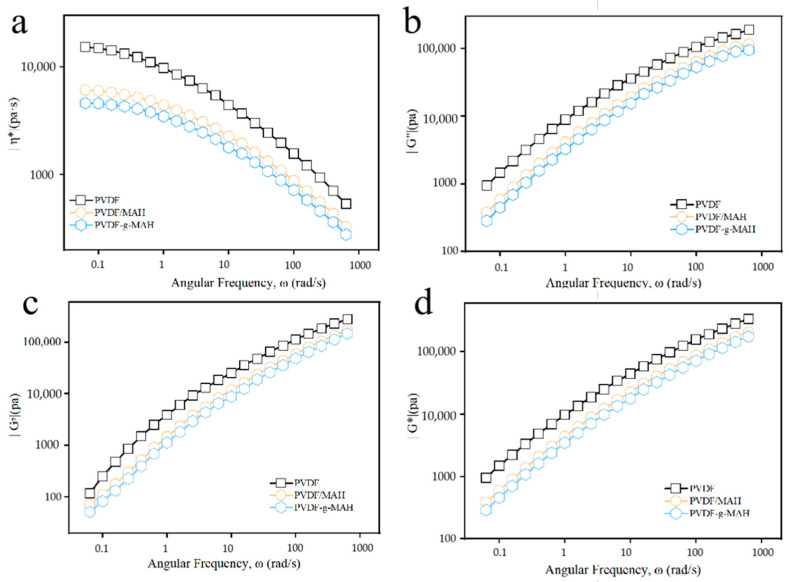
Rheological properties of PVDF, PVDF/MAH and PVDF-g-MAH (G = 0.63%): (**a**), viscosity; (**b**), viscosity modulus; (**c**), elasticity modulus; (**d**), compiex modulus.

Usually, small molecules generally act as diluents and plasticizers of polymers [8]. As shown in Figure 4a, the blending of MAH and PVDF reduces the complex viscosity of PVDF. Compared with that of PVDF/MAH, the complex viscosity of PVDF-g-MAH decreases more obviously. As expected, the complex modulus and viscosity modulus are also decreased with the blending of MAH or the grafting of MAH, indicating that MAH has a plasticizing effect on PVDF.

### 2.5. Mechanical Properties of PVDF, PVDF/MAH and PVDF-g-MAH

**Figure 5:**
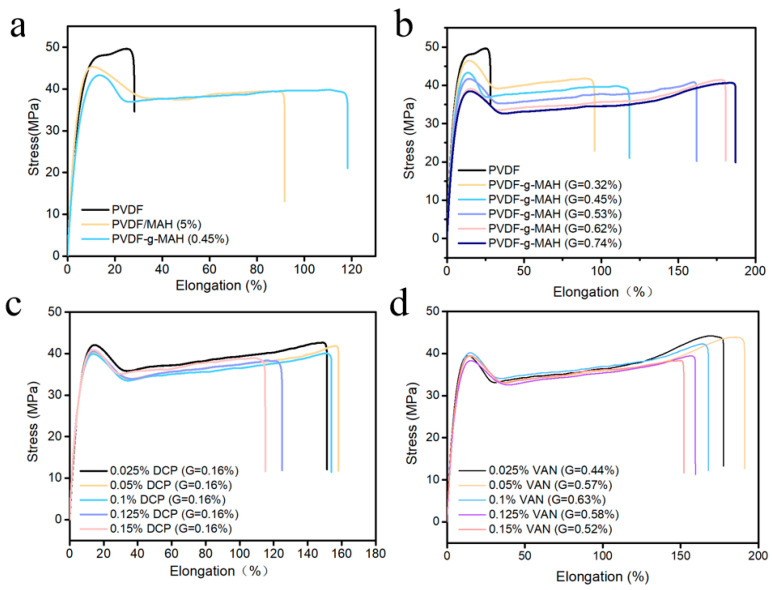
Mechanical properties of PVDF, PVDF/MAH and PVDF-g-MAH: (**a**), Comparison of tensile properties between PVDF, PVDF/MAH and PVDF-g-MAH; (**b**), comparison of tensile properties of grafts with different initial amounts of MAH; (**c**), comparison of tensile properties of grafts with different initial amounts of DCP; (**d**), comparison of tensile properties of grafts with different initial amounts of VAN.

The effect of MAH on the mechanical properties was studied using tensile tests. The yield strength and elongation of pure PVDF, PVDF/MAH and PVDF-g-MAH are shown in Figure 5a. Due to the plastic effect of MAH, the addition of MAH gives rise to a slight reduction in the yield strength and modulus. However, the elongation of PVAD/MAH and PVDF-g-MAH increases significantly, especially for PVDF-g-MAH, indicating that the incorporation of MAH improves the ductility of PVDF. As expected, the higher grafting MAH level leads to the higher elongation at break (Figure 5b–d). Moreover, as shown in Figure 5c,d, the elongation rate of PVDF-g-MAH decreased again with the increasing amount of initiator and VAN used. This may be because as the amount of initiator and VAN increases, PVDF tends to break its chains and thus reduces its elongation rate.

### 2.6. Analysis of the Crystallization and Melting Behavior

**Figure 6:**
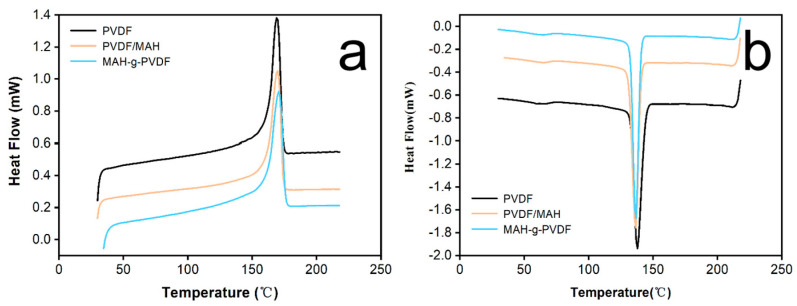
DSC curves of pure PVDF, PVDF/MAH and PVDF-g-MAH (G = 0.63%): (**a**), temperature rise curve; (**b**), cooling curve.

Figure 6 shows the effect of MAH co-blending with PVDF and MAH-grafted PVDF on the crystallization and melting behavior of PVDF. As can be seen in Figure 6a, MAH has a slight effect on the melting behavior of PVDF. The melting temperature of PVDF-g-MAH or PVDF/MAH varies very little. During the cooling process, the crystallinity was decreased with the addition of MAH (Figure 6b). It is possible that some forces were formed among the grafted chains of PVDF molecules, which affect the movement of the molecular chains. The crystallinity (χ_c_) of PVDF in all samples was determined using Equation (1), as follows:

(1) $$\chi_{c}=\frac{\Delta H_{m}}{\emptyset \times \Delta H_{m}^{0}}\times 100\%$$

Formula (1): $\Delta H_{m}^{0}$ is the theoretical enthalpy of 100% crystalline polymer matrix (the $\Delta H_{m}^{0}$ for the PVDF is 104.7 J g^−1^) [28], and ⌀ is the weight fraction of the PVDF matrix. The results are shown in Table 1.

### 2.7. Thermal Stability

**Figure 7:**
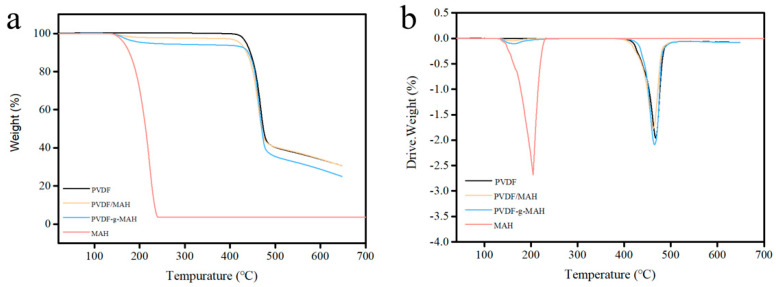
TGA curves of pure PVDF, PVDF/MAH and PVDF-g-MAH (G = 0.63%) under a nitrogen atmosphere: (**a**), TG; (**b**), DTG.

As shown in Figure 7, the thermal stability of PVDF, PVDF/MAH and PVDF-g-MAH was analyzed using TGA under a nitrogen atmosphere. The corresponding data are shown in Table 2. Normally, the initial decomposition temperature of MAH is very low, but pure PVDF has much better thermal stability than MAH [8]. The whole thermal degradation of pure PVDF only has one decomposition process with T_10wt%_ of 444.7 °C, and T_max_ of 467.6 °C. For PVDF/MAH or PVDF-g-MAH, the two separate degradation steps in Figure 7 correspond to the degradation of MAH and PVDF, respectively. Depending on the respective thermal stability, the first degradation step is due to the decomposition of MAH, and PVDF is degraded at higher temperatures. In the degradation process of MAH, the weight loss of MAH in PVDF/MAH and PVDF-g-MAH is 2.5 wt% and 5.7 wt%, respectively, which is lower than the added amount.

### 2.8. Analysis of the Crystallization of PVDF, PVDF/MAH and PVDF-g-MAH

**Figure 8:**
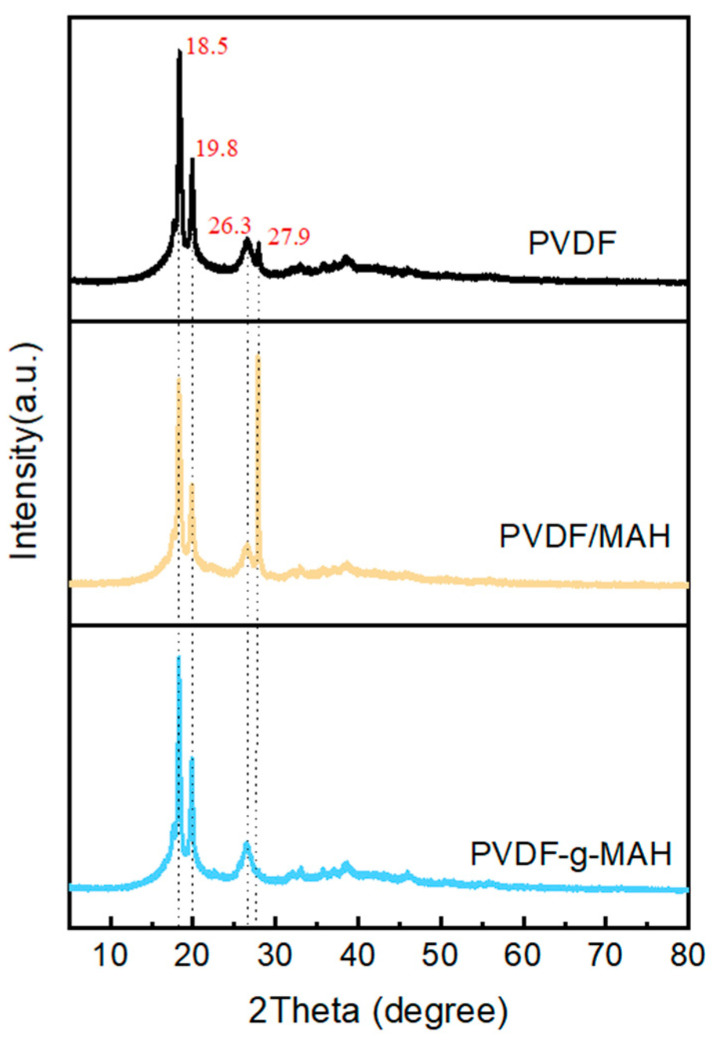
XRD patterns of pure PVDF, PVDF/MAH and PVDF-g-MAH (G = 0.63%).

PVDF is a semi-crystalline polymer containing α, β, and γ crystal phases. Among them, the α phase is the most common and stable crystal type, which usually crystallizes from the melted PVDF [27]. Figure 8 shows the XRD patterns of PVDF, PVDF/MAH and PVDF-g-MAH. It is found that the peaks at 2θ = 18.5, 19.8, 26.3 and 27.9 are the characteristic diffraction peaks of the α phase crystal assigned to (020), (110), (021) and (100) reflections, respectively, indicating that the α phase plays a dominant role. After blending MAH with PVDF, the crystal peak of PVDF/MAH was significantly enhanced at 2θ = 27.9, suggesting that the blending of MAH can promote the crystal growth in (100) direction. However, the crystal peak of PVDF-g-MAH disappears here, demonstrating that g-MAH shows a different effect for the PVDF crystal growth compared to the blending of MAH.

## 3. Materials and Methods

### 3.1. Materials

PVDF was purchased from Hefei economic and technological development zone maga experimental supplies business department (Hefei economic and technological development zone supplies, Hefei, China). MAH, isopropanyl peroxide, sodium hydroxide, hydrochloride, methanol, thymol blue-phenolphthalein indicator and N, N-dimethylformamide (DMF) were purchased from Shanghai Aladdin Biochemical Technology Co., Ltd. (Shanghai, China). Isopropanol alcohol and acetone were purchased from Shanghai Macklin Biochemical Technology Co., Ltd. (Shanghai, China). 9-vinethylanthracene was purchased from Hefei Baierdi Chemical Technology CO., Ltd. (Hefei Baohe District, Hefei, China).

### 3.2. Reactive Processing of PVDF with MAH

PVDF and MAH were first dried in an 80 °C vacuum drying tank for 24 h to remove the water. A certain amount of PVDF, MAH, DCP and VAN were introduced into a torque rheometer. Reactive processing was carried out at 190 °C for 5 min with a rotor speed of 50 rpm. The following Table 3, Table 4 and Table 5 show the specific compositions.

### 3.3. Purification of the Grafted PVDF

The product was dissolved in DMF to form a solution, then the solution was added into large quantities of methanol solvent under vigorous stirring. In this process, unreacted MAH was dissolved in methanol, while the PVDF-g-MAH powder was precipitated. At last, the powder was washed by abundant acetone and methanol, and then dried using vacuum for 24 h.

### 3.4. Determination of the Grafting Degree

The return titration method was used to test the grafting degree of MAH, and thymol blue-phenolphthalein was used as an indicator. The calculation formula is as follows:

(2) $$G=\frac{98.06\times \left(V_{1}-V_{0}\right)\times C}{2\times 100\times m}\times 100\%$$

In Formula (2), G is the grafting degree, m is the relative molecular mass of MAH, V_1_ is the volume of the base consumed by the test sample, V_0_ is the volume of the base consumed by the blank sample, and C is the concentration of isopropanol sodium hydroxide solution [29].

### 3.5. Characterization

A contact angle measuring system (DSA30S) was used to measure the static contact angle between water and PVDF, PVDF/MAH or PVDF-g-MAH.

Differential scanning calorimetry (DSC) (DSC214) was used to measure the melting temperature (Tm), crystallization temperature (Tc), crystallization enthalpy (Hc) and melting enthalpy (Hm). The sample was heated to 250 °C at a heating rate of 10 K/min. After the heat history was removed, the sample was cooled to 30 °C at a cooling rate of 5 K/min, and then heated to 250 °C at a heating rate of 10 K/min.

Fourier transform infrared (FTIR) spectrum analyses (Nicolet.NExus 870FT-IR) were performed on samples pelletized with KBr powder in the range of 4000–400 cm^−1^.

Thermogravimetric analysis (TG) (Labsys Evo) was used to measure the thermogravimetric behavior of the samples in N_2_ atmosphere with a nitrogen flow rate of 20 mL/min. The set temperature range was 25~800 °C, and the heating rate was 10 K/min.

A rotational rheometer (Bohlin Gemini HRnano 200) was used to measure the rheological properties of PVDF, PVDF/MAH and PVDF-g-MAH.

An X-ray diffractometer (XRD) (SmartLab 9 KW) was used to measure the crystal type of PVDF, PVDF/MAH and PVDF-g-MAH. The scanning speed was 2θ = 20°/min.

An electronic universal stretching machine (Instron 5967) was used to measure the mechanical properties of PVDF, PVDF/MAH and PVDF-g-MAH, according to GB/T10403-2006. The elongation rate was 20 mm/min.

## 4. Conclusions

In this work, PVDF-g-MAH was prepared using the simple fusion grafting method. The MAH-grafting degree can be enhanced with an increase in MAH, VAN and DCP contents to a certain extent. Then, the hydrophilicity and mechanical properties of PVDF-g-MAH were studied systematically. Compared to PVDF or PVDF/MAH, PVDF-g-MAH greatly improved the elongation at break of PVDF. More importantly, the hydrophilicity of PVDF was greatly improved, which can extend the application fields of PVDF.

## Figures and Tables

**Table 1:** DSC parameters for PVDF, PVDF/MAH and PVDF-g-MAH composites.

Sample	Tm (°C)	Tc (°C)	∆*H_m_* (J·g^−1^)	Χc (%)
PVDF	170.145	137.718	53.43	51.03
PVDF/MAH	170.581	136.463	51.41	49.1
PVDF-g-MAH	169.552	136.526	49.16	46.9

**Table 2:** TGA data for PVDF, PVDF/MAH and PVDF-g-MAH.

Sample	T_0_ (°C)	T_10wt%_ (°C)	T_50wt%_ (°C)	T_max_ (°C)
PVDF	406.4	444.7	473.7	467.6
PVDF/MAH	133.8	436.7	469.8	462.2
PVDF-g-MAH	132.9	440.5	470.1	465.2
MAH	130.7	173.5	212.9	202.3

**Table 3:** A list of the specific compositions used in blending with different MAH contents.

Name	Dose 1	Dose 2	Dose 3	Dose 4	Dose 5
PVDF	97%	95%	92%	90%	88%
MAH	3%	5%	8%	10%	12%
DCP	0.1%	0.1%	0.1%	0.1%	0.1%
VAN	0.05%	0.05%	0.05%	0.05%	0.05%
G	0.32%	0.45%	0.53%	0.62%	0.74%

**Table 4:** A list of the specific compositions used in blending with different DCP contents.

Name	Dose 1	Dose 2	Dose 3	Dose 4	Dose 5
PVDF	95%	95%	95%	95%	95%
MAH	5%	5%	5%	5%	5%
DCP	0.025%	0.05%	0.1%	0.125%	0.15%
VAN	0.05%	0.05%	0.05%	0.05%	0.05%
G	0.16%	0.28%	0.47%	0.49%	0.43%

**Table 5:** A list of the specific compositions used in blending with different VAN contents.

Name	Dose 1	Dose 2	Dose 3	Dose 4	Dose 5
PVDF	95%	95%	95%	95%	95%
MAH	5%	5%	5%	5%	5%
DCP	0.1%	0.1%	0.1%	0.1%	0.1%
VAN	0.025%	0.05%	0.1%	0.125%	0.15%
G	0.37%	0.45%	0.63%	0.58%	0.52%

## Data Availability

The data presented in this study are available on request from the corresponding author.

## Sample Availability

Samples of the compounds are available from the authors.

## References

[1] Ma H., Yang Y. (2008). Rheology, morphology and mechanical properties of compatibilized poly(vinylidene fluoride) (PVDF)/thermoplastic polyurethane (TPU) blends. Polym. Test..

[2] Iezzi R.A., Gaboury S., Wood K. (2000). Acrylic-fluoropolymer mixtures and their use in coatings. Prog. Org. Coat..

[3] Tian B., Wang X.-Y., Zhang L.-N., Shi F.-N., Zhang Y., Li S.-X. (2016). Preparation of PVDF anionic exchange membrane by chemical grafting of GMA onto PVDF macromolecule. Solid State Ion..

[4] Li W., Li H., Zhang Y.-M. (2009). Preparation and investigation of PVDF/PMMA/TiO2 composite film. J. Mater. Sci..

[5] Kumar C., Viswanath P. (2017). Structure, morphology and wettability studies on Langmuir-Schaefer multilayer of poly(vinylidene fluoride)/poly(methyl methacrylate) blends. Eur. Polym. J..

[6] Liu F., Hashim N.A., Liu Y., Abed M.R.M., Li K. (2011). Progress in the production and modification of PVDF membranes. J. Membr. Sci..

[7] Martins P., Lopes A.C., Lanceros-Mendez S. (2014). Electroactive phases of poly(vinylidene fluoride): Determination, processing and applications. Prog. Polym. Sci..

[8] Chen Z., Pei J., Li R. (2017). Study of the Preparation and Dielectric Property of PP/SMA/PVDF Blend Material. Appl. Sci..

[9] Ye C., Yu Q., He T., Shen J., Li Y., Li J. (2019). Physical and Rheological Properties of Maleic Anhydride-Incorporated PVDF: Does MAH Act as a Physical Crosslinking Point for PVDF Molecular Chains?. ACS Omega.

[10] Naruke A., Liang X., Nakajima K., Nishi T. (2022). Morphological characterization of the novel fine structure of the PMMA/PVDF blend. Polym. J..

[11] Tang Y.P., Cai T., Loh D., O’Brien G.S., Chung T.S. (2017). Construction of antifouling lumen surface on a poly(vinylidene fluoride) hollow fiber membrane via a zwitterionic graft copolymerization strategy. Sep. Purif. Technol..

[12] Ameduri B. (2022). Copolymers of vinylidene fluoride with functional comonomers and applications therefrom: Recent developments, challenges and future trends. Prog. Polym. Sci..

[13] Dang Z.-M., Yan W.-T., Xu H.-P. (2007). Novel high-dielectric-permittivity poly(vinylidene fluoride)/polypropylene blend composites: The influence of the poly(vinylidene fluoride) concentration and compatibilizer. J. Appl. Polym. Sci..

[14] Govinna N., Sadeghi I., Asatekin A., Cebe P. (2019). Thermal properties and structure of electrospun blends of PVDF with a fluorinated copolymer. J. Polym. Sci. Part B Polym. Phys..

[15] Behera K., Chen J.-F., Yang J.-M., Chang Y.-H., Chiu F.-C. (2023). Evident improvement in burning anti-dripping performance, ductility and electrical conductivity of PLA/PVDF/PMMA ternary blend-based nanocomposites with additions of carbon nanotubes and organoclay. Compos. Part B.

[16] Shi Z., Zhao G., Zhang L., Wang G. (2022). Lightweight, strong, flame-retardant PVDF/PMMA microcellular foams for thermal insulation fabricated by supercritical CO2 foaming. Compos. Part B.

[17] Ma L., Liu C., Dou R., Yin B. (2021). Significantly improved high dielectric MWCNTs filled PVDF/PS/HDPE composites via constructing double bi-continuous structure. Compos. Part B.

[18] Ameduri B. (2009). From vinylidene fluoride (VDF) to the applications of VDF-containing polymers and copolymers: Recent developments and future trends. Chem. Rev..

[19] Qiu G.M., Zhu L.P., Zhu B.K., Xu Y.Y., Qiu G.L. (2008). Grafting of styrene/maleic anhydride copolymer onto PVDF membrane by supercritical carbon dioxide: Preparation, characterization and biocompatibility. J. Supercrit. Fluids.

[20] Qiu J., Zhao L., Zhai M., Ni J., Zhou H., Peng J., Wei G. (2008). Pre-irradiation grafting of styrene and maleic anhydride onto PVDF membrane and subsequent sulfonation for application in vanadium redox batteries. J. Power Sources.

[21] Iqbal M., Chuai C., Huang Y., Che C. (2010). Modification of low-density polyethylene by graft copolymerization with maleic anhydride and blends with polyamide 6. J. Appl. Polym. Sci..

[22] Ismail H., Mathialagan M. (2013). Compatibilization of bentonite filled ethylene-propylene-diene monomer composites: Effect of maleic anhydride grafted EPDM. J. Appl. Polym. Sci..

[23] Choi E.Y., Kim C.K., Park C.B. (2022). Fabrication of MA-EPDM grafted MWCNTs by reactive extrusion for enhanced interfacial adhesion and mechanical properties of PP/MA-EPDM composite. Compos. Part B.

[24] Simonetti P., Nazir R., Gooneie A., Lehner S., Jovic M., Salmeia K.A., Gaan S. (2019). Michael addition in reactive extrusion: A facile sustainable route to developing phosphorus based flame retardant materials. Compos. Part B.

[25] Liu Y., Liu S., Liu Z., Lei Y., Jiang S., Zhang K., Yu J. (2021). Enhanced mechanical and biodegradable properties of PBAT/lignin composites via silane grafting and reactive extrusion. Compos. Part B.

[26] Bonnet A., Ramfel B., Chopinez F., Triballier K., Werth M., Pascal T. (2005). Process for Grafting Fluoropolymer and Multilayered Structure Containing Obtained Graft Polymer. FR. CN1572822. https://kns.cnki.net/kns8/defaultresult/index.

[27] Gregorio R., de Souza Nociti N.C.P. (1995). Effect of PMMA addition on the solution crystallization of the alpha and beta phases of poly(vinylidene fluoride) (PVDF). J. Phys. D Appl. Phys..

[28] Mohamadi M. (2022). Interpretation of thermal transitions and phase transformations in semi-crystalline PVDF/PEO/graphene nanocomposites characterized by modulated-temperature DSC. J. Therm. Anal. Calorim..

[29] Xiao X.-X., Zhang X.-H. (2007). The grafting rate of PP-g-MAH was determined by the back-titration method. Friends Chem. Ind..

